# Impact of concordant ICU triage on hospital mortality: a nationwide retrospective multilevel analysis

**DOI:** 10.3389/fmed.2026.1785019

**Published:** 2026-03-12

**Authors:** Ruoxu Dou, Jinghong Xu, Ka Yin Lui, Xiaodong Song, Xiaoguang Hu, Yanping Zhu, Changjie Cai

**Affiliations:** 1 Department of Critical Care, The First Affiliated Hospital of Sun Yat-Sen University, Guangzhou, China; 2 Department of Anesthesiology, The First Affiliated Hospital of Sun Yat-Sen University, Guangzhou, China

**Keywords:** critical care, ICU triage, intensive care units, multilevel model, specialization

## Abstract

**Background:**

Specialty intensive care units (ICUs) provide diagnosis-specific patient care. Nonetheless, the advantage or disadvantage of ICU specialization remains debatable. We conducted a multicenter retrospective cohort study to evaluate the clinical outcomes of specialty ICUs in critical care.

**Methods:**

This retrospective study reviewed adult ICU patients in the eICU Collaborative Research Database (eICU-CRD), a United States (US)-based multicentered ICU cohort database. Patients over 16 years old with documented Acute Physiology and Chronic Health Evaluation (APACHE) IV scores were enrolled and grouped by their admitted ICUs. Multilevel logistic regression models were performed to assess the relationships between hospital mortality and ICU type/ICU triage conditions in patients admitted with a diagnosis of cerebrovascular accident (CVA), acute coronary syndrome (ACS), coronary artery bypass graft (CABG), pneumonia (PNA) and other respiratory diseases, and trauma. A parallel multiple mediation analysis was performed to examine whether interventions in the ICU mediated the association between ICU triage and hospital mortality.

**Results:**

A total of 136,236 patients admitted to 271 ICUs from 186 hospitals were enrolled. In multilevel analysis, admission to cardiac ICU (OR: 0.81, *p* < 0.001) and surgical ICU (OR: 0.88, *p* = 0.041) were associated with lower mortality risks, and admission to Neuro-ICU (OR: 1.32, *p* < 0.001) was linked to a higher risk of mortality compared to general ICU in total cohort. In patients without trauma, general ICU or discordant triage was not associated with a higher in-hospital mortality risk than concordant ICU triage. In trauma patients, discordant ICU triage was associated with higher hospital mortality (OR: 1.87, *p* = 0.005), partially mediated by mechanical ventilation (OR = 1.007, *p* < 0.001).

**Conclusion:**

Specialized concordant ICUs were not associated with lower hospital mortality risk compared to general ICUs after adjusting for confounders. Under resource-constrained settings, discordant ICU triage may be a viable option.

## 1 Introduction

Over the recent three decades, intensive care medicine has become a featured specialty of clinical medicine, along with the increase in the number of intensive care units (ICUs) and their capacity to carry out critical care services to meet the mounting demand. It is reported that the ICU-to-hospital-bed ratio in the United States has increased from 13.5 to 16.2% in the first decade of the 21st century (1, 2). The capacity and expertise of ICUs to diagnose and manage critical illnesses have now been widely regarded as an indicator of the overall service quality of a hospital. Therefore, establishing and optimizing the critical care system under limited medical resources for the best treatment outcomes has become a significant challenge in hospital management. One of the most discussed trends is the specialization of ICUs. A specialized ICU, namely, cardiology ICU, medical ICU, neurology ICU, etc., refers to an ICU where a designated service specialty concentrates its medical resources and provides diagnosis-specific care for selected patients within the scope of its expertise (3). Specialized ICUs manage a narrower spectrum of diseases, thus they may better understand patients’ primary conditions and disease progression, and be able to implement more tailored monitoring and therapy, intuitively improving the clinical outcomes (4). It was found that the mortality rate of patients with cerebral hemorrhage treated in the Neuro-ICU was significantly lower than in general ICU (5–7). Some similar results have been shown in earlier studies of admitted to specialty care units for shock, cardiovascular diseases (7–10). On the contrary, in a retrospective cohort study with a larger sample (84,182 patients), Lott et al. demonstrated no advantage from an ideal (“diagnosis-appropriate”) specialty ICU over general ICU in improving risk-adjusted survival or shortening ICU length of stay (LOS) (11). As comorbidities and multiple organ failure are commonly presented in critically ill patients, general ICU may be better at balancing the risk and benefit of various treatments, distinguishing primary and secondary diagnoses, and overseeing the big picture of an ICU encounter. As the average age of ICU patients continues to rise and multimorbidity become more prevalent (12), there is an urgent need to discuss whether general ICUs facilitate a more integrated and comprehensive approach to managing complex, multi-system failures.

The previous studies focused on specific specialty ICUs and a few medical conditions, limiting their conclusions to a particular field. Therefore, we aim to first conduct a comprehensive analysis of ICU patients from a large multicenter database with a wide range of diagnoses who were admitted to various specialty ICUs. Secondly, to investigate the effect of a concordant ICU triage versus a discordant ICU specialty or general ICU on in-hospital mortality risk, we selected five specialized diseases with relatively high case volumes across each specialized ICU for subgroup analysis, including CVA (cerebrovascular accident), ACS (acute coronary syndrome), pneumonia and other respiratory disease, CABG (coronary artery bypass graft), and trauma.

## 2 Methods

### 2.1 Data source

The retrospective cohort study was based on data from the eICU Collaborative Research Database (eICU-CRD), a multi-center intensive care unit database with high granularity de-identified health data for over 200,000 ICU admissions across the United States between 2014 and 2015 (13). The up-to-date version 2.0 of eICU-CRD was employed. After completing the Collaborative Institutional Training Initiative training course “Data or Specimens Only Research,” our group gained access to the eICU-CRD (certification number: 2093226) and was approved by the Massachusetts Institute of Technology Affiliates to employ the above data. Informed consent was not applicable since the patient’s identity had been de-identified.

### 2.2 Study population and data extraction

All patients 16 years and older with documented APACHE IV scores and admission diagnosis in the eICU database were enrolled in the study (14). Exclusion criteria were not the first admission. Patients were stratified based on their initially-admitted ICU specialty, including general ICU (GICU), surgical ICU (SICU), medical ICU (MICU), neurological ICU (Neuro-ICU), and CICU, which consists of cardiac ICU, CCU-CTICU (Coronary Care Unit/Cardiothoracic Intensive Care Unit), CSICU (Cardiac Surgical Intensive Care Unit), and CTICU (Cardiothoracic Intensive Care Unit).

The demographic and clinical data, including sex, age, ethnicity, APACHE-IV score, diagnosis, and comorbidities, were retrieved by PostgreSQL (version 9.6; PostgreSQL Global Development Group). The implementation of ICU interventions (mechanical ventilation, dialysis, vasopressor drug usage, and antibiotics use) was used to evaluate the demand for aggressive medical service. Hospital-level covariates, including geographic region, teaching status, and bed capacity, were also collected. No missing values were present in the variables included in this study; therefore, no imputation was performed.

The study focused on a primary outcome of in-hospital mortality.

### 2.3 Statistical analysis

Baseline demographic and clinical features were individually compared between different ICU units. Pearson’s chi-square test was performed for categorical data, and continuous data were compared by pairwise Student’s *t*-test or one-way analysis of variance (ANOVA) (parametric data) across groups, or Wilcoxon rank-sum test or Mann–Whitney *U*-test (non-parametric data).

We employed multilevel mixed-effects logistic regression models to compare the in-hospital mortality risks across different ICU types. We adjusted for the fixed effects of confounders such as gender, age, APACHE-IV score, comorbidities (hypertension, diabetes, CKD, COPD, heart failure, cancer), and hospital characteristics (teaching status, bed capacity compared to beds ≥500), and the random effects at the hospital level to account for between-hospital variability.

Given the significant heterogeneity in admission diagnoses across ICU types, we further focused on five sub-cohorts that were diagnosed with the following common conditions at admission: CVA (cerebrovascular accident), ACS (acute coronary syndrome), pneumonia and other respiratory disease, CABG (coronary artery bypass graft), and trauma. We aimed to test whether the initial ICU specialty, that is, a concordant versus discordant ICU triage, was associated with distinct in-hospital mortality risks for these highly specialized conditions. Consequently, we re-classified their initially-admitted ICU specialty into concordant ICU triage, discordant ICU triage, and general ICU according to the consistency between admission diagnosis and ICU specialty. The ICU triage grouping details are displayed in Table 1.

**Table 1:** Cohort re-grouping based on ICU triage.

Diseases	Concordant ICU triage	Discordant ICU triage	General ICU
CVA	Neuro-ICU	CICU, MICU, SICU	GICU
ACS	CICU	Neuro-ICU, MICU, SICU	GICU
CABG	CICU	Neuro-ICU, MICU, SICU	GICU
PNA/Other respiratory diseases	MICU	Neuro-ICU, CICU, SICU	GICU
Trauma	SICU	CICU, MICU, Neuro-ICU	GICU

CVA, cerebrovascular accident; ACS, acute coronary syndrome; CABG, coronary artery bypass graft; PNA, pneumonia; CICU, cardiac ICU; GICU, general ICU; MICU, medical ICU; Neuro ICU, neurology ICU; SICU, surgical ICU.

To describe causal assumptions among ICU types, hospital mortality, confounders, and mediators, we constructed a directed acyclic graph (directed acyclic graphs) following the latest guidelines (15). A parallel multiple mediation analysis was performed to examine whether ICU interventions (including mechanical ventilation, vasopressor use, dialysis, and antibiotic therapy) mediated the association between ICU triage and hospital mortality. To determine effect sizes and obtain 95% confidence intervals, we assessed direct and indirect effects using the bootstrap method with 1,000 iterations. R Package “bruceR” was used in analysis, which estimates all mediation paths simultaneously while controlling for potential confounders. For clinical interpretability, regression coefficients from logistic regression models were converted to odds ratios (ORs) with 95% confidence intervals (CIs) using the formula OR = exp(*β*). This analysis was restricted to diagnostic subgroups in which ICU triage was significantly associated with hospital mortality in multilevel mixed-effects models accounting for hospital-level random effects.

The above analyses were computed with results visualized by R (version 4.4.3, R Foundation for Statistical Computing, Vienna, Austria).^1^ A two-tailed *p*-value <0.05 was considered statistically significant.

## 3 Results

### 3.1 Overview of ICU settings

**Figure 1:**
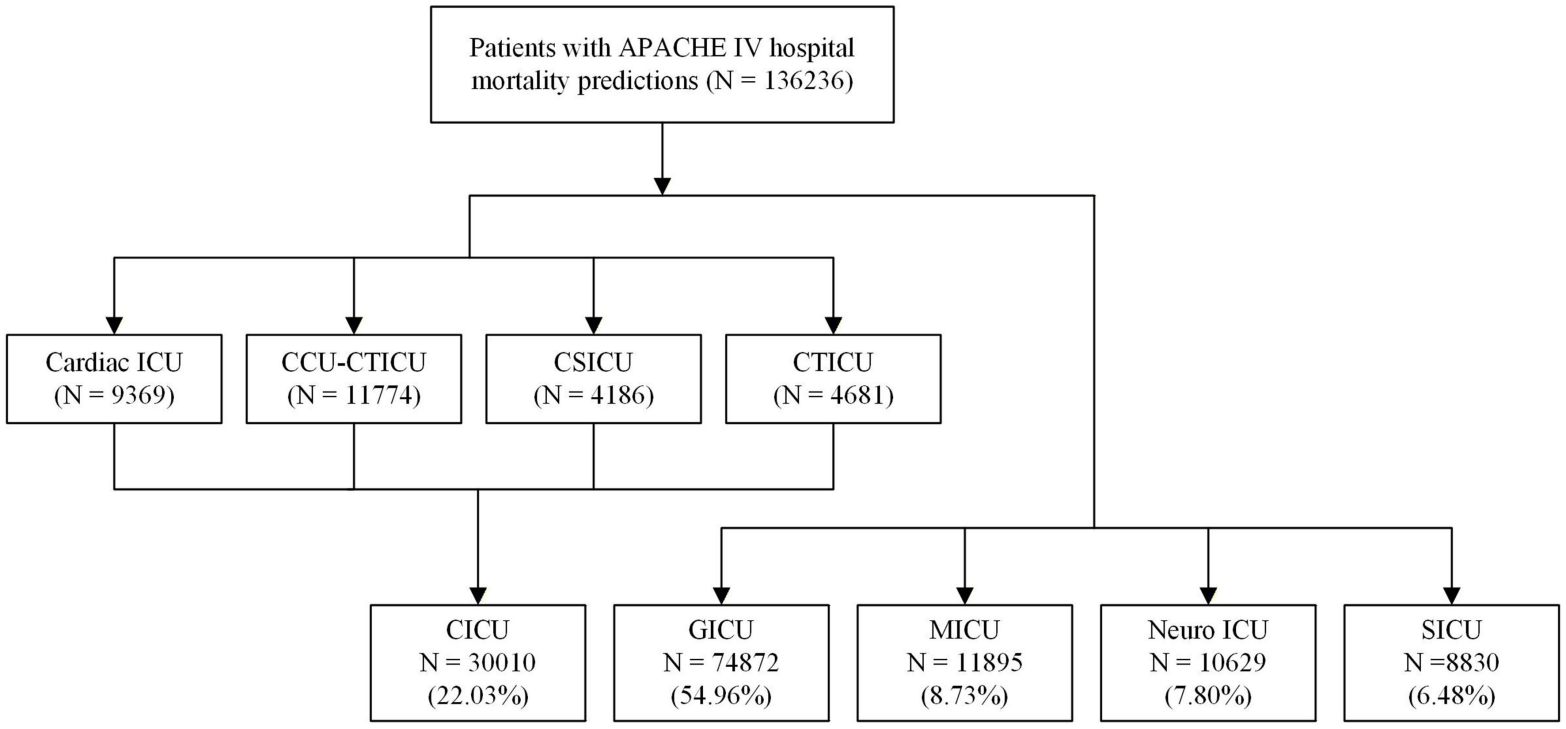
Flowchart of study cohort selection.

The participant enrollment flowchart is illustrated in Figure 1. This study included 136,236 patients with documented APACHE IV scores from 271 ICUs across 190 hospitals in the eICU database. More than 50% of patients were admitted to general ICUs (GICU, 54.96%), followed by cardiac ICUs (CICU, 22.03%), medical ICUs (MICU, 8.73%), neurology ICUs (neuro ICU, 7.80%), and surgical ICUs (SICU, 6.48%).

**Figure 2:**
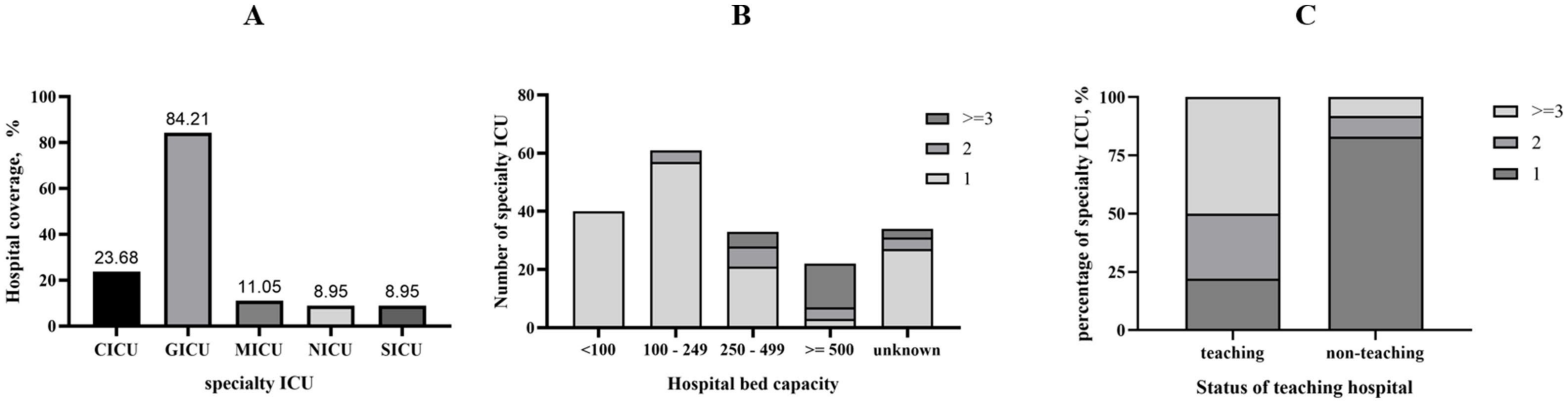
Overview of ICU settings. **(A)** The coverage ratio of specialty ICUs in US hospitals; **(B)** the number of ICU types in hospitals with different bed capacities; **(C)** the number of ICU types in teaching or non-teaching hospitals.

Of the 192 hospitals, 160 (84.21%) had a GICU (84.21%), followed by CICU in 23.68% of hospitals. The number of ICU types was associated with both the hospital’s bed capacity and its status as a teaching hospital. All hospitals with fewer than 100 beds hosted only one ICU specialty, but over half (68.18%) of the hospitals with more than 500 beds had three or more ICU specialties. Besides, nearly 75% of teaching hospitals had two or more ICUs, compared to less than 25% in non-teaching hospitals (Figure 2).

### 3.2 Patient characteristics

Baseline characteristics of the total cohort are summarized in Table 2. The overall cohort had a median age of 65 years (IQR: 53–76), 54% male, and a median APACHE IV score of 51 (IQR: 37–68). APACHE IV scores varied significantly by ICU type, with the lowest in Neuro-ICU (43, IQR: 31–58) and the highest in MICU (56, IQR: 41–75) (*p* < 0.001). ICU admission diagnoses included sepsis (13%), CVA (7%), cardiac arrest (7%), ACS (6%), and gastrointestinal bleeding (5%), with significant different distribution across ICU types (*p* < 0.001). Comorbidities varied by ICU type, with Neuro-ICU patients exhibiting the fewest. Cancer prevalence was highest in SICU (10% vs. 5% in GICU, *p* < 0.001). In ICU interventions, mechanical ventilation, dialysis, vasopressors, and antibiotics were administered more frequently in MICU than in Neuro-ICU (*p* < 0.001). The overall ICU mortality rate was 6%, highest in MICU (8% vs. 4% in neuro ICU, *p* < 0.001), and the median ICU length of stay was 42 h (IQR: 23–76), shortest in GICU (41 h, IQR: 22–73). Hospital characteristics analysis showed that 27% of patients were admitted to teaching hospitals (59% in neuro ICU vs. 16% in GICU), while 39% were treated in large hospitals with ≥500 beds (79% in neuro ICU vs. 20% in GICU).

**Table 2:** Demographic, clinical, and admitted hospital information characteristics of patients.

Variables	Total	GICU	CICU	MICU	Neuro ICU	SICU	*p*-value
*n*	136,236	74,872	30,010	11,895	10,629	8,830	
Age	65 (53, 76)	65 (52, 76)	66 (55, 76)	65 (52, 76)	63 (50, 75)	64 (52, 75)	<0.001
Gender, male	73,611 (54)	69,472 (54)	17,493 (58)	6,182 (52)	5,481 (52)	5,011 (57)	<0.001
APACHE IV	51 (37, 68)	51 (37, 69)	50 (38, 67)	56 (41, 75)	43 (31, 58)	50 (37, 67)	<0.001
Admission diagnosis							<0.001
ACS	8,343 (6)	3,434 (5)	4,289 (14)	339 (3)	71 (1)	210 (2)	
ARF	1932 (1)	1,305 (2)	273 (1)	265 (2)	15 (0)	74 (1)	
Asthma	3,948 (3)	2,561 (3)	687 (2)	512 (4)	65 (1)	123 (1)	
CABG	4,771 (4)	1,324 (2)	3,044 (10)	1 (0)	5 (0)	397 (4)	
Cardiac arrest	9,135 (7)	4,833 (6)	3,158 (11)	731 (6)	136 (1)	277 (3)	
Chest pain unknown	761 (1)	407 (1)	295 (1)	36 (0)	5 (0)	18 (0)	
CHF	5,592 (4)	3,006 (4)	1844 (6)	491 (4)	64 (1)	187 (2)	
Coma	2082 (2)	1,358 (2)	265 (1)	233 (2)	147 (1)	79 (1)	
CVA	9,758 (7)	4,202 (6)	865 (3)	552 (5)	3,475 (33)	664 (8)	
Other CV	3,593 (3)	1,393 (2)	1,607 (5)	250 (2)	167 (2)	176 (2)	
DKA	4,384 (3)	3,157 (4)	503 (2)	562 (5)	54 (1)	108 (1)	
GI bleed	7,277 (5)	4,768 (6)	885 (3)	968 (8)	116 (1)	540 (6)	
GI obstruction	1,232 (1)	823 (1)	114 (0)	72 (1)	15 (0)	208 (2)	
Neuro	4,640 (3)	2028 (3)	330 (1)	276 (2)	1804 (17)	202 (2)	
Overdose	4,268 (3)	3,073 (4)	528 (2)	485 (4)	97 (1)	85 (1)	
PNA	4,577 (3)	3,180 (4)	639 (2)	583 (5)	69 (1)	106 (1)	
Other respiratory diseases	7,970 (6)	4,726 (6)	1,266 (4)	1,309 (11)	229 (2)	440 (5)	
Sepsis	18,087 (13)	12,119 (16)	2,554 (9)	2,436 (20)	306 (3)	672 (8)	
Trauma	5,884 (4)	2,972 (4)	289 (1)	154 (1)	1,376 (13)	1,093 (12)	
Valvular disorders	2,795 (2)	697 (1)	1921 (6)	0 (0)	2 (0)	175 (2)	
Other	25,207 (19)	13,506 (18)	4,654 (16)	1,640 (14)	2,411 (23)	2,996 (34)	
Comorbidities							
Hypertension	19,878 (15)	10,837 (14)	5,135 (17)	1,057 (9)	1,644 (15)	1,205 (14)	<0.001
Diabetes	15,623 (11)	9,486 (13)	3,625 (12)	1,151 (10)	636 (6)	725 (8)	<0.001
CKD	11,869 (9)	6,508 (9)	2,869 (10)	1,282 (11)	426 (4)	784 (9)	<0.001
COPD	10,544 (8)	6,822 (9)	2030 (7)	1,039 (9)	257 (2)	396 (4)	<0.001
Heart failure	11,361 (8)	6,522 (9)	3,202 (11)	994 (8)	209 (2)	434 (5)	<0.001
Cancer	7,336 (5)	4,080 (5)	992 (3)	564 (5)	783 (7)	917 (10)	<0.001
ICU intervention							
Mechanical ventilation	41,293 (30)	22,150 (30)	9,838 (33)	4,148 (35)	2,218 (21)	2,939 (33)	<0.001
Dialysis	5,722 (4)	3,251 (4)	1,215 (4)	775 (7)	146 (1)	335 (4)	<0.001
Vasopressor drug usage	16,227 (12)	8,489 (11)	4,581 (15)	1,602 (13)	502 (5)	1,053 (12)	<0.001
Antibiotics use	32,640 (24)	20,695 (28)	6,173 (21)	2,716 (23)	1,195 (11)	1861 (21)	<0.001
Outcomes							
Hospital death	12,061 (8.9)	6,650 (8.9)	2,384 (7.9)	1,458 (12.3)	849 (8)	720 (8.2)	<0.001
ICU death	7,619 (6)	4,204 (6)	1,601 (5)	898 (8)	463 (4)	453 (5)	<0.001
ICU LOS, hours	42 (23, 76)	41 (22, 73)	43 (24, 76)	46 (25, 88)	44 (24, 87)	46 (25, 89)	<0.001
Hospital information							
Bed							<0.001
<100	8,072 (6)	6,966 (9)	567 (2)	192 (2)	0 (0)	347 (4)	
≥500	52,508 (39)	14,795 (20)	15,607 (52)	7,584 (64)	8,384 (79)	6,138 (70)	
100–249	29,199 (21)	22,945 (31)	4,185 (14)	993 (8)	652 (6)	424 (5)	
250–499	32,177 (24)	22,209 (30)	6,388 (21)	1,507 (13)	631 (6)	1,442 (16)	
Unknown	14,280 (10)	7,957 (11)	3,263 (11)	1,619 (14)	962 (9)	479 (5)	
Teaching status	37,355 (27)	12,040 (16)	10,622 (35)	4,296 (36)	6,231 (59)	4,166 (47)	< 0.001

Data are presented as number (percentage) or median (interquartile range). APACHE IV, Acute Physiology and Chronic Health Evaluation IV; ACS, acute coronary syndrome; ARF, acute renal failure; CABG, Coronary Artery Bypass Graft; CHF, Congestive heart failure; CVA, Cerebrovascular accident; CV, Cerebrovascular; DKA, Diabetic Ketoacidosis; GI, Gastrointestinal; PNA, pneumonia; CKD, Chronic kidney disease; COPD, Chronic Obstructive Pulmonary Disease; GICU, Medical-Surgical ICU; MICU, Medical ICU; Neuro ICU, Neurology ICU; SICU, Surgical ICU; LOS, length of stay.

### 3.3 Multilevel models of hospital mortality ICU admission patients

**Figure 3:**
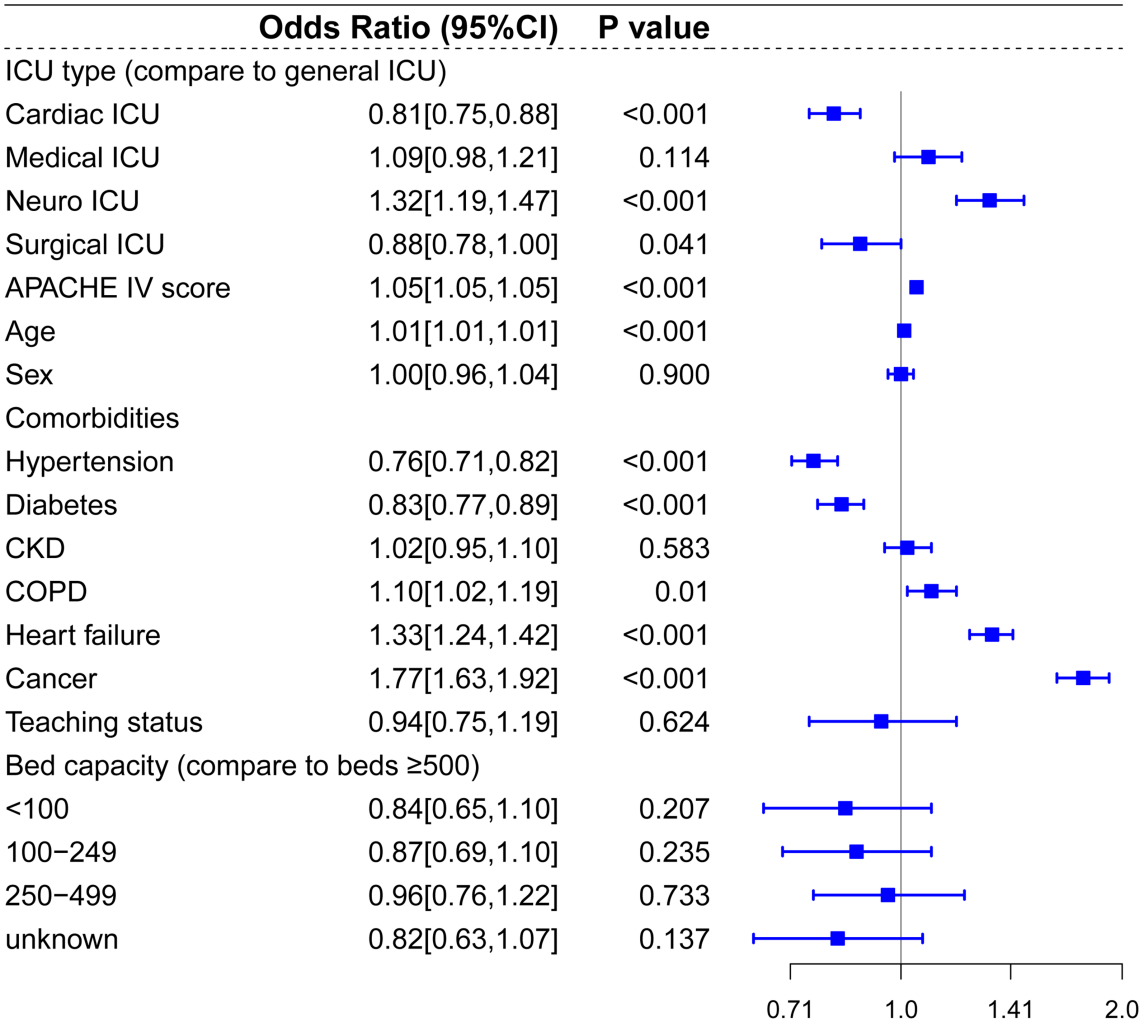
Multilevel logistic regression model for risk of death in the total cohort.

Our multilevel mixed-effects logistics regression model identified the following independent predictors for increased hospital mortality risks among all enrolled participants: admission to Neuro-ICU (odds ratio, OR: 1.32, 95% confidence interval, CI: 1.19–1.47, *p* < 0.001), APACHE-IV score (OR: 1.05, 95%CI: 1.05–1.05, *p* < 0.001), age (OR: 1.011, 95% CI: 1.01–1.01, *p* < 0.001), COPD (OR: 1.10, 95%CI: 1.02–1.19, *p* = 0.01), heart failure (OR: 1.3, 95%CI: 1.24–1.42, *p* < 0.001), cancer (OR: 1.77, 95%CI: 1.63–1.92, *p* < 0.001). However, admission to cardiac ICU (OR: 0.81, 95%CI: 0.75–0.88, *p* < 0.001) and surgical ICU (OR: 0.88, 95% CI: 0.78–1.00, *p* = 0.041) were associated with lower mortality risks compared to general ICU. (Figure 3).

### 3.4 Subgroup analysis of five common specialized conditions

**Figure 4:**
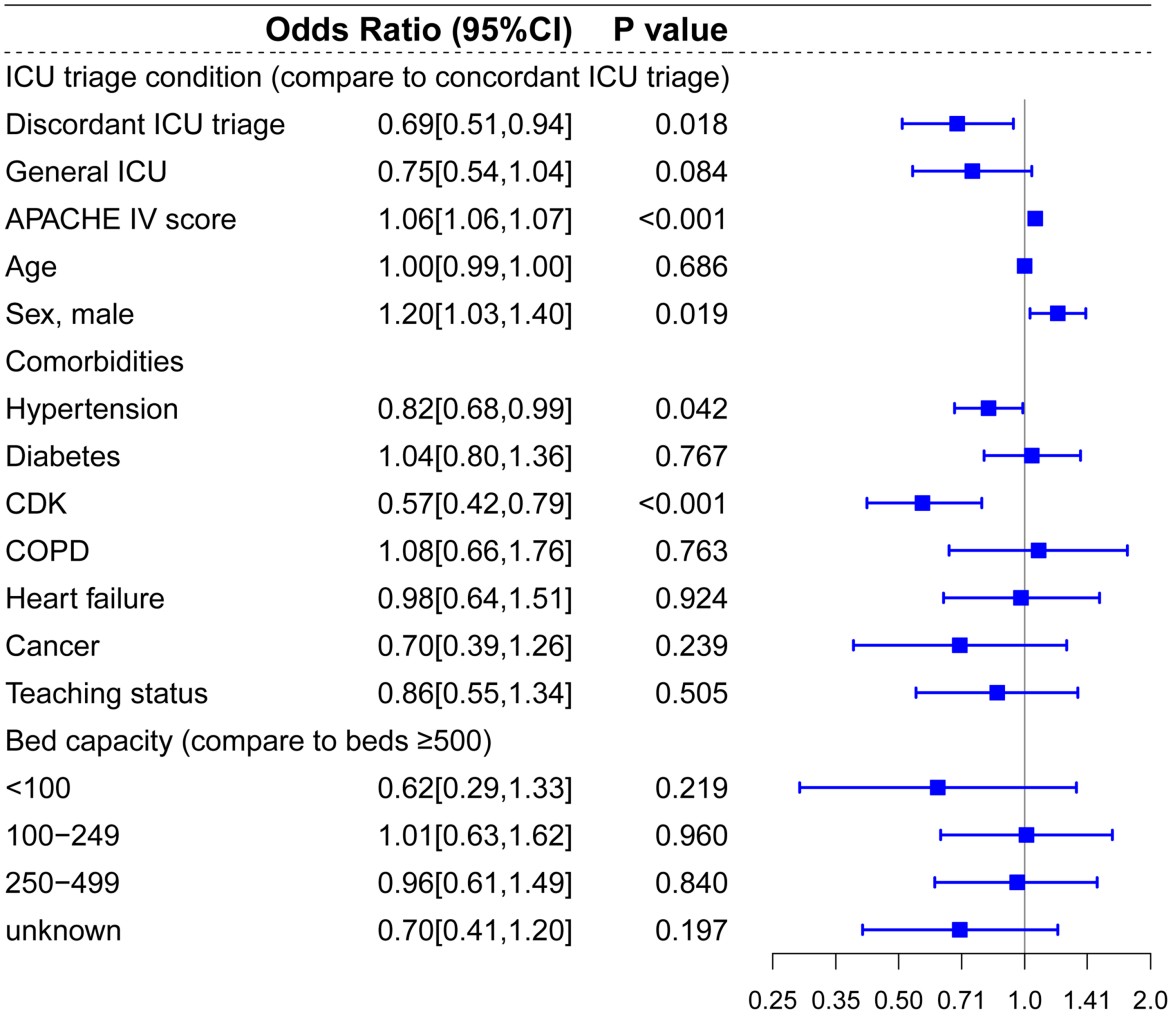
Multilevel logistic regression model for risk of death in patients diagnosed with CVA.

**Figure 5:**
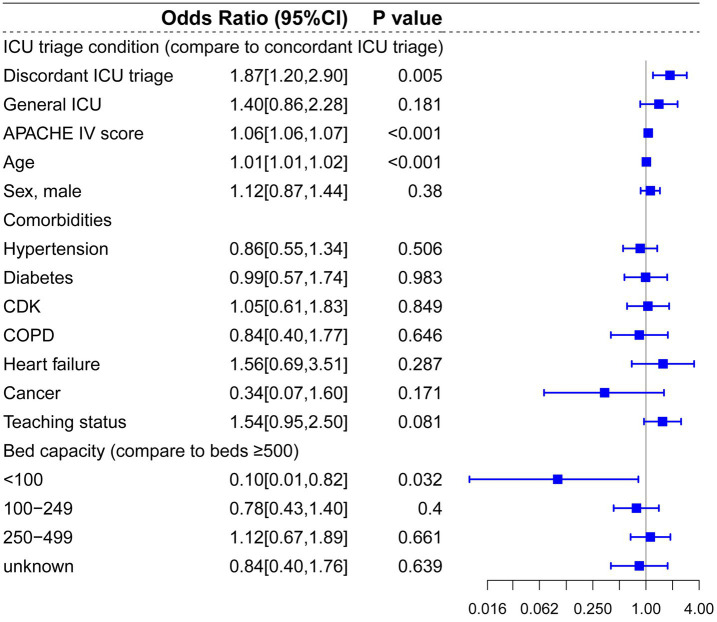
Multilevel logistic regression model for risk of death in patients diagnosed with trauma.

Next, we conducted subgroup analysis on five common conditions that might benefit from treatment in a concordant, specialized ICU using multilevel mixed-effects regression models, including CVA, ACS, CABG, trauma, PNA and other respiratory diseases. In patients diagnosed with CVA at admission, discordant ICU triage was associated with lower hospital mortality compared to concordant ICU triage (OR: 0.69, 95%CI: 0.51–0.94, *p* = 0.018) (Figure 4). Contrarily, patients diagnosed with trauma who were initially admitted to a discordant ICU triage showed significantly higher in-hospital mortality risks compared to a concordant ICU specialty (OR: 1.87, 95%CI: 1.2–2.9, *p* = 0.005) (Figure 5).

**Figure 6:**
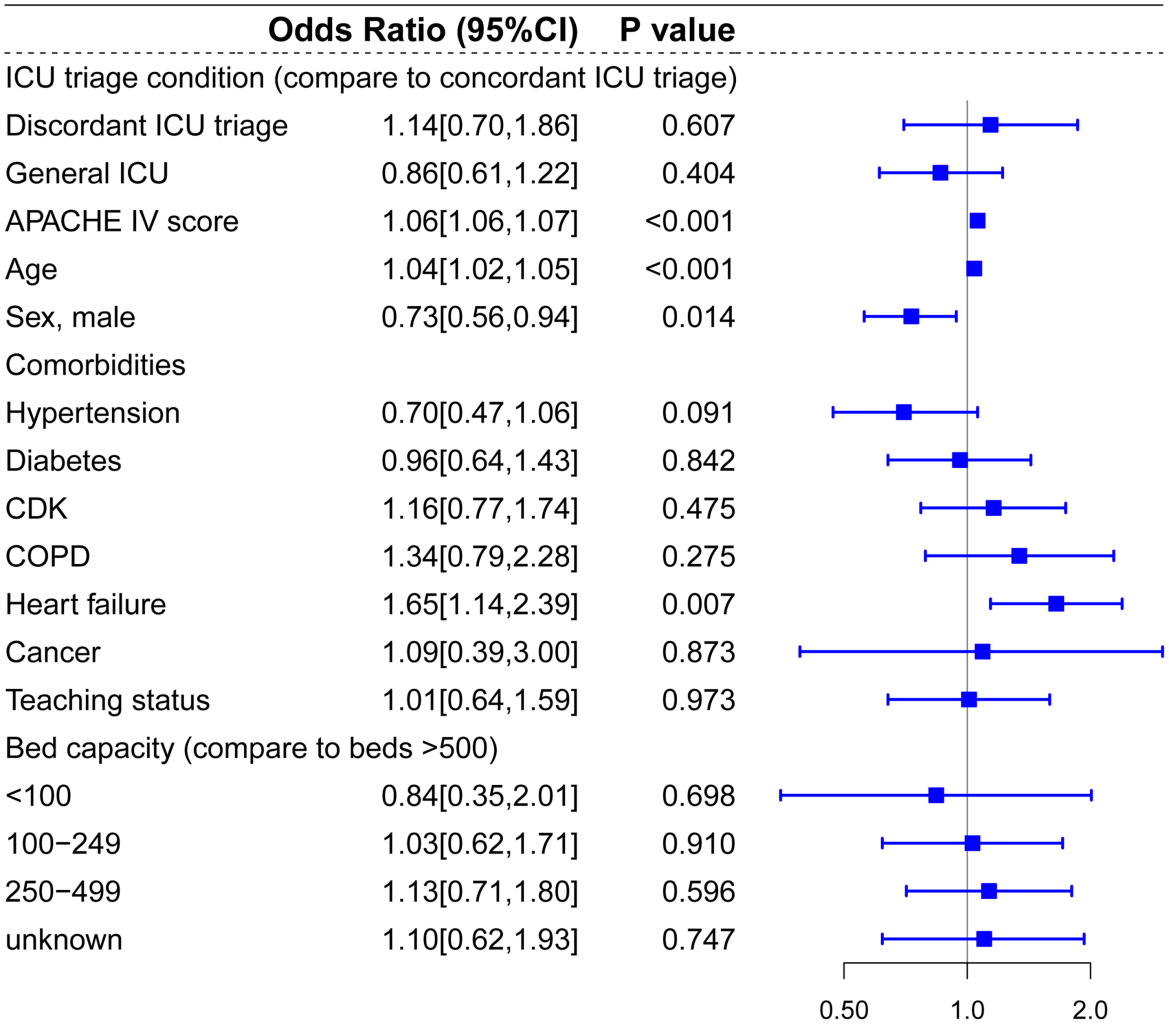
Multilevel logistic regression model for risk of death in patients diagnosed with ACS.

**Figure 7:**
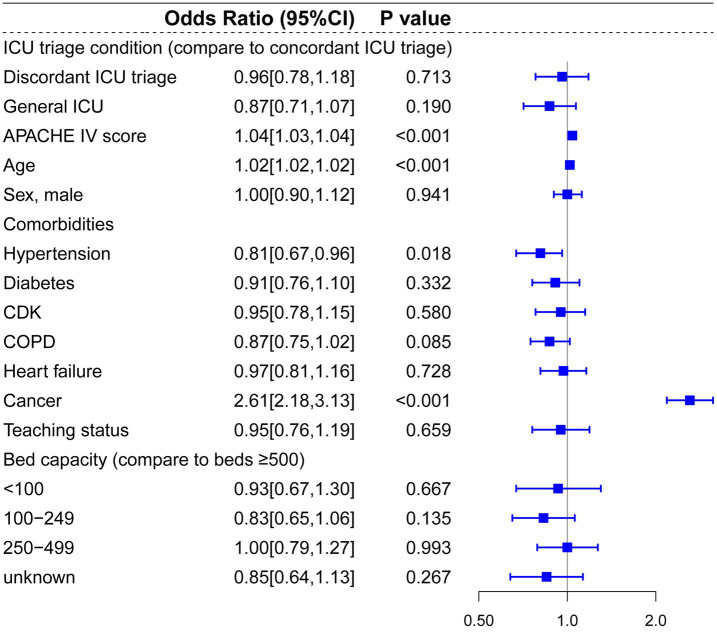
Multilevel logistic regression model for risk of death in patients diagnosed with PNA and other respiratory diseases.

**Figure 8:**
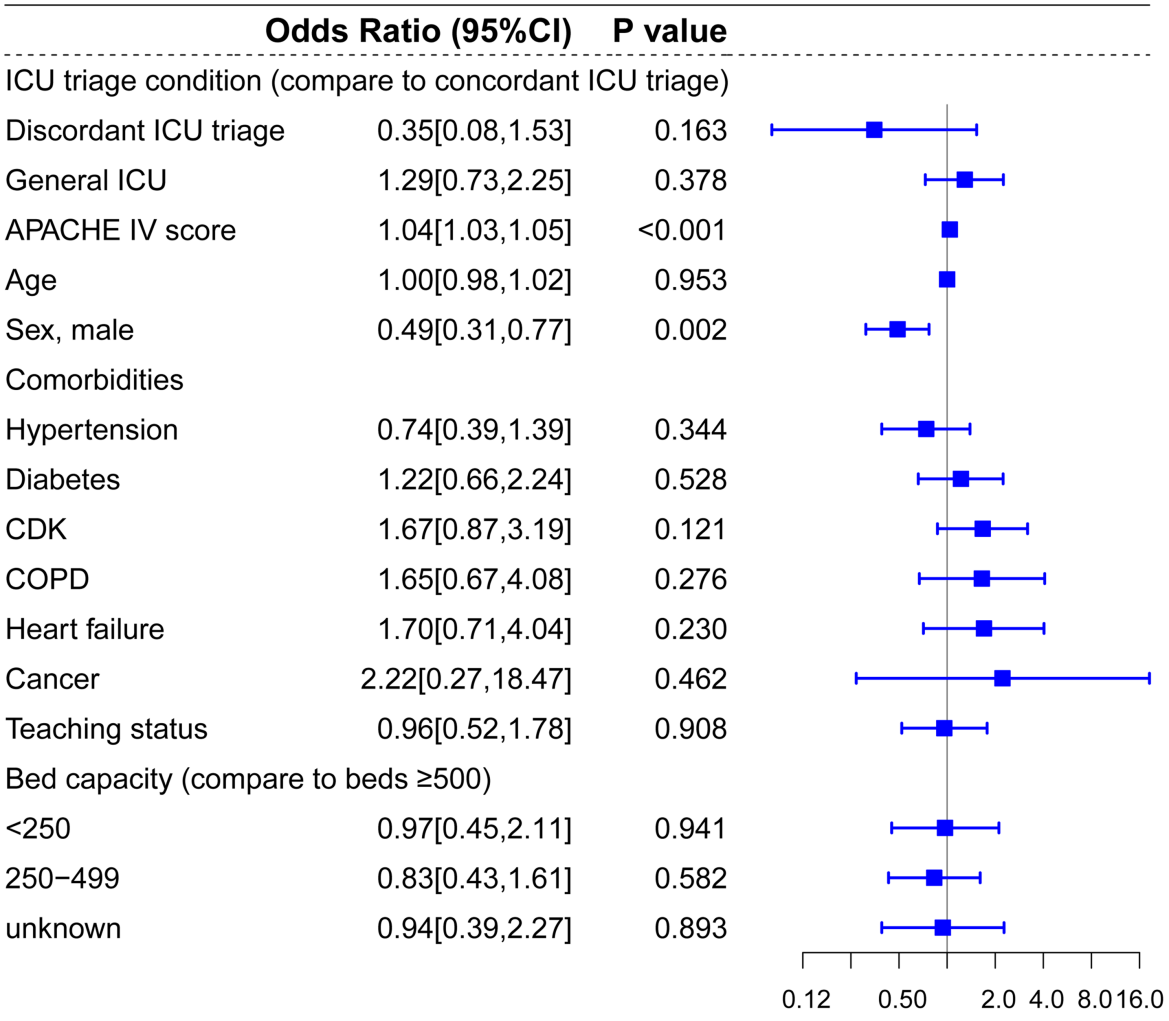
Multilevel logistic regression model for risk of death in patients diagnosed with CABG.

Regarding patients diagnosed with ACS, pneumonia and other respiratory diseases, and CABG, there were no significant statistical differences between concordant ICU triage, discordant ICU triage, and general ICU (Figures 6, 7, 8). APACHE-IV score and age were generally associated with a higher hospital mortality across all diseases.

### 3.5 Mediation analysis of CVA and trauma patient

**Figure 9:**
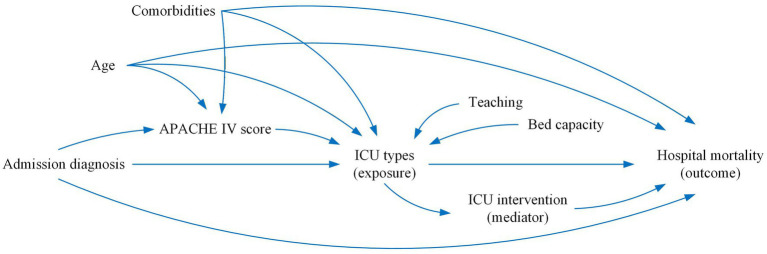
DAG of with exposure to ICU types, outcome of hospital mortality, confounder, and mediators.

**Figure 10:**
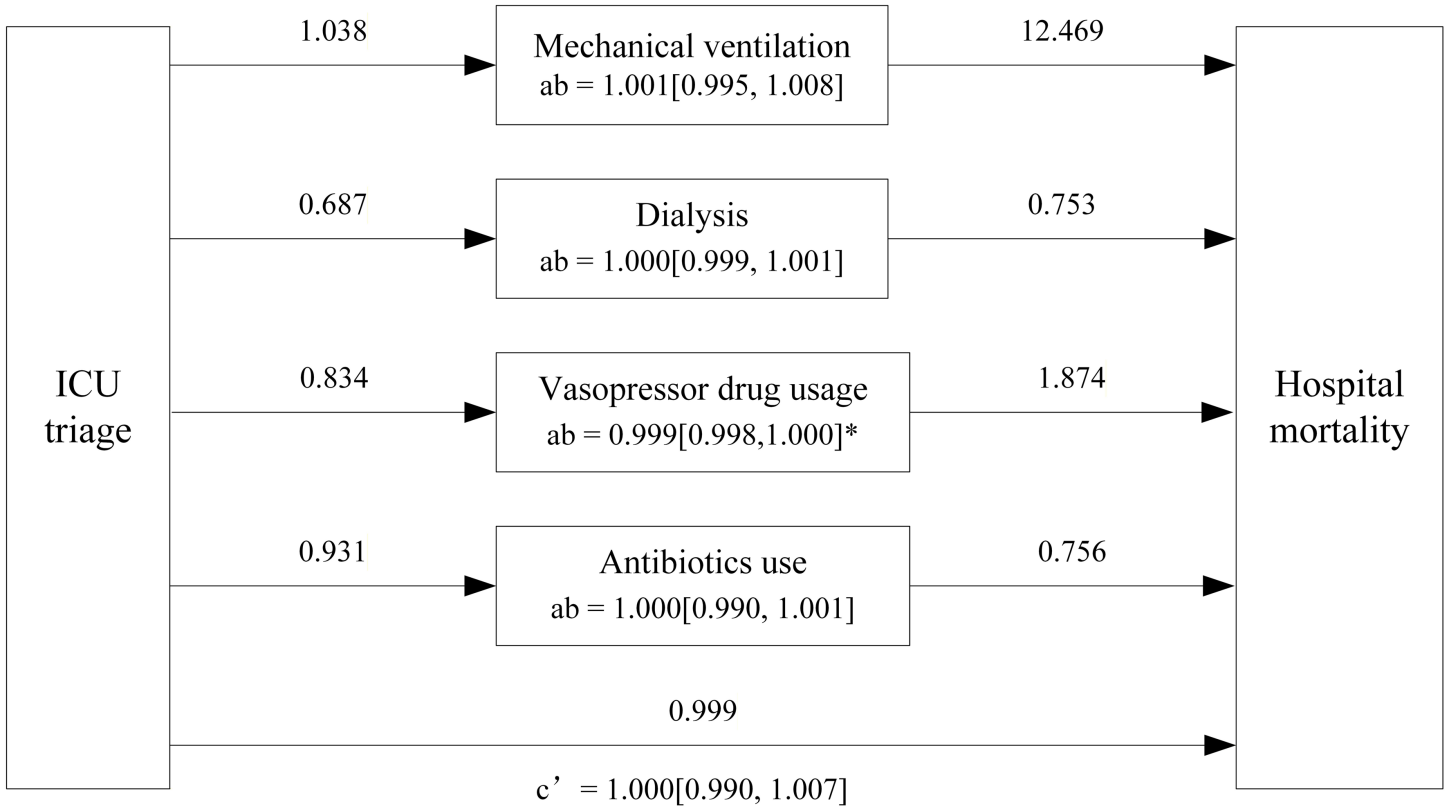
Mediation effects of ICU triage on hospital mortality in CVA subgroup. The figure illustrates the mediation analysis with ICU triage as the independent variable, and hospital mortality as the dependent variable. The numbers above the arrows indicate the odds ratio (OR) values for the respective paths. The indirect effect (ab) is represented by the path from ICU triage (concordant vs. discordant) to hospital mortality through the mediators, ICU interventions. The direct effect (c’) represents the direct relationship between ICU triage and hospital mortality, excluding the mediators. The values in brackets [] indicate the Bootstrap 95% Confidence Interval (CI) for each effect OR values. * *p* < 0.5, ** *p* < 0.01, *** *p* < 0.001.

**Figure 11:**
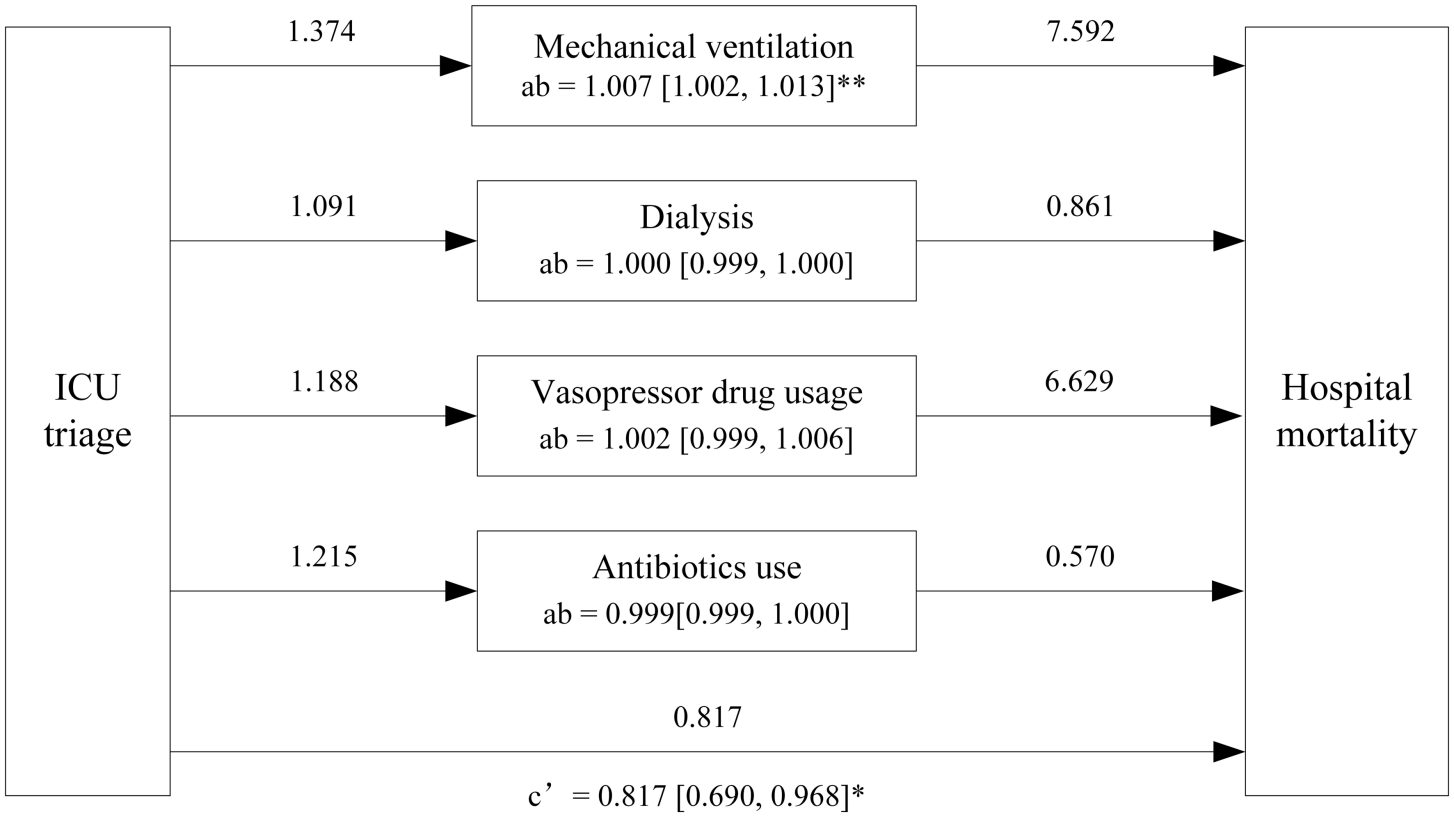
Mediation effects of ICU triage on hospital mortality in trauma subgroup. The figure illustrates the mediation analysis with ICU triage as the independent variable, and hospital mortality as the dependent variable. The numbers above the arrows indicate the odds ratio (OR) values for the respective paths. The indirect effect (ab) is represented by the path from ICU triage (concordant vs. discordant) to hospital mortality through the mediators, ICU interventions. The direct effect (c’) represents the direct relationship between ICU triage and hospital mortality, excluding the mediators. The values in brackets [] indicate the Bootstrap 95% Confidence Interval (CI) for each effect OR values. * *p* < 0.5, ** *p* < 0.01, *** *p* < 0.001.

Mediation analysis confirmed the mediating effect relationship between hospital mortality, ICU types and ICU intervention, including mechanical ventilation, dialysis, vasopressor drug usage and Antibiotics use. In the trauma subgroup, ICU triage demonstrated a significant direct effect on hospital mortality (OR = 0.817, 95% CI: 0.690–0.968; *p* = 0.02), whereas no significant direct effect was observed in the CVA subgroup (OR = 1.000, 95% CI: 0.990 to 1.007; *p* = 0.87). Regarding indirect effects, vasopressor use significantly mediated the association between ICU triage and hospital mortality in the CVA subgroup (OR = 0.999, 95% CI: 0.998 to 1.000; *p* = 0.029). In contrast, in the trauma subgroup, mechanical ventilation showed a significant mediating effect, with ICU triage associated with reduced hospital mortality through increased use of mechanical ventilation (OR = 1.007, 95% CI: 1.002 to 1.013; *p* < 0.001). No statistically significant indirect effects were observed for dialysis or antibiotic use in either the CVA or trauma subgroups, and vasopressor use did not demonstrate a significant mediating effect in the trauma subgroup (*p* > 0.05) (Figures 9–11).

## 4 Discussion

Despite being widely discussed, no consensus was reached on the benefit of ICU specialization. In our study, which included a large cohort of ICU patients who were managed at different levels of hospitals, demonstrated that specialized ICU was not associated with lower hospital mortality risk compared to the general ICU after adjusting for confounders, in fact, Neuro-ICU showed an increased in-hospital mortality risk. Regarding specialized conditions, CVA patients who were initially admitted to a concordant ICU (Neuro-ICU) were not linked with lower mortality risk, while trauma patients admitted to a concordant ICU (SICU) demonstrated lower mortality risk.

This study outlines characteristics of participating hospitals, including bed capacity, teaching status, and ICU unit number. Notably, general ICUs were widely implemented across institutions, while the establishment of specialized ICUs appeared correlated with bed capacity≥300 and academic affiliation. Our findings align with existing evidence suggesting that tertiary care centers and teaching hospitals, which typically possess greater infrastructural resources and organizational maturity, are more likely to adopt specialized ICUs. Furthermore, large medical complexes often implement critical care organizations (CCOs) to coordinate multiple ICUs, a strategy shown to enhance both operational efficiency and cost-effectiveness through centralized governance and resource optimization (16–19).

Compared to prior studies, the present research encompassed a more extensive selection of specialized ICUs and for multi-level hierarchical data, multilevel models effectively control for random effects at the hospital level. In the multilevel analysis including all patients, bed capacity (compared to bed >500) and hospitals’ teaching status had no significant effect on hospital mortality. Admission to cardiac ICU (OR: 0.81, *p* < 0.001) and surgical ICU (OR: 0.88, *p* = 0.041) were associated with lower mortality risks, and admission to Neuro-ICU (OR: 1.32, *p* < 0.001) was linked to a higher risk of mortality compared to general ICU. In the subgroup and mediation analyses, discordant ICU triage was associated with lower in-hospital mortality in the CVA group (OR: 0.69, *p* = 0.018), and this relationship appears to be mediated by the use of vasopressors (OR = 0.999, 95% CI: 0.998 to 1.000; *p* = 0.029). In contrast, in the trauma group, concordant ICU triage was associated with lower in-hospital mortality (OR: 1.87, *p* = 0.005), which was partially mediated by mechanical ventilation (indirect effect = −0.019, *p* < 0.001). These findings indicate that the impact of different ICU types on mortality is associated with interventions within the ICU. In baseline analysis (Supplementary Tables 2–6), Neuro-ICUs exhibited lower rates of organ support therapy, potentially reflecting their greater focus on specialized conditions (neurological disorders). However, this specialization may also imply reduced or delayed interventions for other complications, thereby influencing mortality rates. The higher utilization of life-support interventions like mechanical ventilation in SICUs compared to discordant ICUs may correlate with lower mortality rates, particularly for trauma patients who often require more advanced life support. It should be noted that discordant screening often involves selection bias, as patients with more severe CVAs (such as those with significant cerebral infarction or cerebral edema) may be more likely to be transferred to the neurocritical care unit for specialized treatment, thereby influencing in-hospital mortality rates.

Recent advancements in evidence-based critical care guidelines and bundled management protocols have enhanced comprehensive patient management in ICUs (20–22). Critically ill patients often develop multi-organ complications, including infections, shock, AKI, and ARDS. This requires the specialty ICU team to acquire more skills and knowledge in different specialties. And it makes the boundaries between specialty and general ICUs gradually blurred (23). General ICUs demonstrate superior resource efficiency through optimized monitoring-intervention workflows, particularly in multi-organ dysfunction management (11). In contrast, specialty ICUs may lead to resource redundancy due to focusing on specialized diseases, especially when admit non-ideal patients, resource utilization efficiency will be further reduced (3, 11). At the same time, specialty ICUs may provide more detailed assessment and monitoring of the corresponding specialty disease. This improves diagnostic accuracy, but may also prolong ICU LOS (24). Therefore, tertiary hospitals require specialty ICUs for academic and operational needs. However, physician training and resource allocation should balance specialty-specific competencies with universal critical care principles. Interdepartmental collaboration must be prioritized to enhance clinical adaptability and establish synergistic resource-sharing mechanisms between general and specialty ICUs.

Several factors limited the present study. Despite utilizing multilevel models to mitigate the impact of random effects at the hospital level, the study subjects still exhibit a certain degree of heterogeneity due to the inherent limitations of retrospective research. Our study based on only one country’s current situation of ICUs. The research conclusions may vary depending on the medical, economic, dietary, and ethnic characteristics of different countries and regions. As the data for this study were sourced from publicly available databases in developed countries, the findings may be more applicable to resource-rich nations capable of establishing specialized ICUs. We also suggest that in resource-limited scenarios, discordant triage may be an acceptable option without compromising patient safety. The follow-up research may need to collect detailed clinical data from more countries and areas to obtain research results that are further consistent with the real world. In addition, to avoid the heterogeneity of the research population, we also need to focus on particular patient populations to verify the conclusions for specific specialties.

## 5 Conclusion

Our study demonstrates that specialized concordant ICU care does not associate with an in-hospital survival advantage over general ICU care after adjusting for confounders. Consequently, discordant triage may be acceptable in resource-limited environments without compromising patient safety. However, trauma patients derived a survival benefit from concordant triage, potentially mediated by differences in the mechanical ventilation application. These findings further emphasize the importance of prioritizing specialized trauma centers in medical resource planning. Future prospective studies are needed to determine if specific patient subgroups might still benefit from specialized triage.

## Data Availability

Publicly available datasets were analyzed in this study. This data can be found at: https://eicu-crd.mit.edu/gettingstarted/access/.

## Footnotes

1
https://www.R-project.org/

## Glossary

ICUs: Intensive care units
eICU-CRD: eICU Collaborative Research Database
US: United States
APACHE: Acute Physiology and Chronic Health Evaluation
GICU: general ICU
AKI: acute kidney injury
CVA: cerebrovascular accident
ACS: acute coronary syndrome
CICU: cardiology ICU
MICU: medical ICU
Neuro ICU: neurology ICU
LOC: length of stay
SICU: surgery ICU
CTICU: cardiothoracic ICU
CSICU: cardiac surgical ICU
COPD: chronic obstructive pulmonary disease
ANOVA: one-way analysis of variance
CCO: critical care organization
ARDS: acute respiratory distress syndrome
CABG: coronary artery bypass graft
