# Supplementary material for: Impact of concordant ICU triage on hospital mortality: a nationwide retrospective multilevel analysis
Source: Front Med (Lausanne). 2026 Mar 12;13:1785019. doi: 10.3389/fmed.2026.1785019 (PMC13018113; doi:10.3389/fmed.2026.1785019)
Supplement: Supplementary file 1 [file Data_Sheet_1.docx]

**Supplementary Table 1. Demographic, clinical, and admitted hospital information characteristics of patients in different CICUs.**

| Variables | Total | Cardiac ICU | CCU-CTICU | CSICU | CTICU | p |
| --- | --- | --- | --- | --- | --- | --- |
| n | 30010 | 9369 | 11774 | 4186 | 4681 |  |
| Age | 66 (55, 76) | 65 (53, 76) | 66 (55, 76) | 69 (59, 77) | 66 (56, 75) | < 0.001 |
| Gender, male | 17493 (58) | 5119 (55) | 6970 (59) | 2480 (59) | 2924 (62) | < 0.001 |
| Ethnicity |  |  |  |  |  | < 0.001 |
| African American | 3918 (13) | 1615 (17) | 1248 (11) | 200 (5) | 855 (18) |  |
| Asian | 372 (1) | 103 (1) | 132 (1) | 47 (1) | 90 (2) |  |
| Caucasian | 23175 (77) | 6702 (72) | 9702 (82) | 3617 (86) | 3154 (67) |  |
| Hispanic | 1125 (4) | 546 (6) | 194 (2) | 80 (2) | 305 (7) |  |
| Native American | 96 (0) | 9 (0) | 21 (0) | 53 (1) | 13 (0) |  |
| Other/Unknown | 1324 (4) | 394 (4) | 477 (4) | 189 (5) | 264 (6) |  |
| APACHE IV | 50 (38, 67) | 50 (37, 68) | 49 (37, 64) | 52 (40, 70) | 50 (39, 66) | < 0.001 |
| Admission diagnosis |  |  |  |  |  | < 0.001 |
| ACS | 4289 (14) | 1354 (14) | 2170 (18) | 475 (11) | 290 (6) |  |
| ARF | 273 (1) | 138 (1) | 99 (1) | 17 (0) | 19 (0) |  |
| Asthma | 687 (2) | 312 (3) | 278 (2) | 64 (2) | 33 (1) |  |
| CABG | 3044 (10) | 61 (1) | 1127 (10) | 576 (14) | 1280 (27) |  |
| Cardiac arrest | 3158 (11) | 1189 (13) | 1401 (12) | 380 (9) | 188 (4) |  |
| Chest pain unknown | 295 (1) | 117 (1) | 128 (1) | 20 (0) | 30 (1) |  |
| CHF | 1844 (6) | 771 (8) | 713 (6) | 174 (4) | 186 (4) |  |
| Coma | 265 (1) | 132 (1) | 95 (1) | 19 (0) | 19 (0) |  |
| CVA | 865 (3) | 453 (5) | 276 (2) | 49 (1) | 87 (2) |  |
| Other CV | 1607 (5) | 246 (3) | 747 (6) | 227 (5) | 387 (8) |  |
| DKA | 503 (2) | 219 (2) | 197 (2) | 59 (1) | 28 (1) |  |
| GI bleed | 885 (3) | 393 (4) | 339 (3) | 79 (2) | 74 (2) |  |
| GI obstruction | 114 (0) | 69 (1) | 14 (0) | 18 (0) | 13 (0) |  |
| Neuro | 330 (1) | 206 (2) | 87 (1) | 10 (0) | 27 (1) |  |
| Overdose | 528 (2) | 281 (3) | 190 (2) | 49 (1) | 8 (0) |  |
| Pneumonia | 639 (2) | 342 (4) | 196 (2) | 63 (2) | 38 (1) |  |
| Other respiratory diseases | 1266 (4) | 579 (6) | 471 (4) | 110 (3) | 106 (2) |  |
| Sepsis | 2554 (9) | 1110 (12) | 1066 (9) | 252 (6) | 126 (3) |  |
| Trauma | 289 (1) | 142 (2) | 70 (1) | 30 (1) | 47 (1) |  |
| Valvular disorders | 1921 (6) | 6 (0) | 655 (6) | 461 (11) | 799 (17) |  |
| Other | 4654 (16) | 1249 (13) | 1455 (12) | 1054 (25) | 896 (19) |  |
| Comorbidities |  |  |  |  |  |  |
| Hypertension | 5135 (17) | 1986 (21) | 1858 (16) | 102 (2) | 1189 (25) | < 0.001 |
| Diabetes | 3625 (12) | 1434 (15) | 1333 (11) | 38 (1) | 820 (18) | < 0.001 |
| CKD | 2869 (10) | 1088 (12) | 1333 (11) | 70 (2) | 378 (8) | < 0.001 |
| COPD | 2030 (7) | 877 (9) | 828 (7) | 74 (2) | 251 (5) | < 0.001 |
| Heart failure | 3202 (11) | 1323 (14) | 1243 (11) | 228 (5) | 408 (9) | < 0.001 |
| Cancer | 992 (3) | 445 (5) | 315 (3) | 78 (2) | 154 (3) | < 0.001 |
| ICU intervention |  |  |  |  |  |  |
| Ventilation | 9838 (33) | 2612 (28) | 3931 (33) | 1821 (44) | 1474 (31) | < 0.001 |
| Dialysis | 1215 (4) | 519 (6) | 356 (3) | 198 (5) | 142 (3) | < 0.001 |
| Vasopressor use | 4581 (15) | 1113 (12) | 1667 (14) | 1063 (25) | 738 (16) | < 0.001 |
| Antibiotics use | 6173 (21) | 2768 (30) | 2076 (18) | 143 (3) | 1186 (25) | < 0.001 |
| Secondary outcomes |  |  |  |  |  |  |
| ICU death | 1601 (5) | 659 (7) | 564 (5) | 238 (6) | 140 (3) | < 0.001 |
| ICU LOS, hours | 43 (24, 76) | 44 (25, 82) | 43 (25, 77) | 36 (22, 64) | 46 (25, 79) | < 0.001 |
| Hospital information |  |  |  |  |  |  |
| bed |  |  |  |  |  | < 0.001 |
| <100 | 567 (2) | 279 (3) | 288 (2) | 0 (0) | 0 (0) |  |
| >= 500 | 15607 (52) | 3578 (38) | 7224 (61) | 1960 (47) | 2845 (61) |  |
| 100 - 249 | 4185 (14) | 1859 (20) | 1469 (12) | 857 (20) | 0 (0) |  |
| 250 - 499 | 6388 (21) | 2307 (25) | 2204 (19) | 571 (14) | 1306 (28) |  |
| unknown | 3263 (11) | 1346 (14) | 589 (5) | 798 (19) | 530 (11) |  |
| teaching | 10622 (35) | 1595 (17) | 5693 (48) | 1419 (34) | 1915 (41) | < 0.001 |
| region |  |  |  |  |  | < 0.001 |
| Midwest | 10587 (35) | 2311 (25) | 6396 (54) | 839 (20) | 1041 (22) |  |
| Northeast | 1211 (4) | 615 (7) | 596 (5) | 0 (0) | 0 (0) |  |
| South | 798 (3) | 0 (0) | 0 (0) | 798 (19) | 0 (0) |  |
| West | 11164 (37) | 4931 (53) | 2901 (25) | 238 (6) | 3094 (66) |  |
| Other/Unknown | 6250 (21) | 1512 (16) | 1881 (16) | 2311 (55) | 546 (12) |  |

Data are presented as number (percentage) or median (interquartile range).

APACHE IV, Acute Physiology and Chronic Health Evaluation IV; ACS, acute coronary syndrome; ARF, acute renal failure; CABG, Coronary Artery Bypass Graft; CHF, Congestive heart failure; CVA, Cerebrovascular accident; CV, Cerebrovascular; DKA, Diabetic Ketoacidosis; GI, Gastrointestinal; PNA, pneumonia; CKD, Chronic kidney disease; COPD, Chronic Obstructive Pulmonary Disease; GICU, Medical-Surgical ICU; MICU, Medical ICU; Neuro ICU, Neurology ICU; SICU, Surgical ICU; LOS, length of stay.

**Supplementary Table 2. Demographic, clinical, and admitted hospital information characteristics of patients admitted with CVA.**

|  | Overall | Concordant ICU triage | Discordant ICU triage | General ICU | P value |
| --- | --- | --- | --- | --- | --- |
| n | 9758 | 3475 | 2081 | 4202 |  |
| Age | 66.00 [54.00, 78.00] | 66.00 [54.00, 77.00] | 65.00 [54.00, 78.00] | 66.00 [54.00, 79.00] | 0.229 |
| Gender, Male | 4809 (49.28) | 1705 (49.06) | 1004 (48.25) | 2100 (49.98) | 0.413 |
| APACHE IV | 45.00 [34.00, 60.00] | 44.00 [33.00, 60.00] | 46.00 [34.00, 60.00] | 45.00 [34.00, 60.00] | 0.027 |
| Comorbidities |  |  |  |  |  |
| Hypertension | 3708 (38.00) | 826 (23.77) | 1014 (48.73) | 1868 (44.46) | <0.001 |
| Diabetes | 992 (10.17) | 243 (6.99) | 222 (10.67) | 527 (12.54) | <0.001 |
| CKD | 724 (7.42) | 128 (3.68) | 231 (11.10) | 365 (8.69) | <0.001 |
| COPD | 213 (2.18) | 56 (1.61) | 40 (1.92) | 117 (2.78) | 0.001 |
| Heart failure | 308 (3.16) | 60 (1.73) | 77 (3.70) | 171 (4.07) | <0.001 |
| Cancer | 178 (1.82) | 65 (1.87) | 31 (1.49) | 82 (1.95) | 0.423 |
| ICU intervention |  |  |  |  |  |
| Ventilation | 1726 (17.69) | 665 (19.14) | 349 (16.77) | 712 (16.94) | 0.02 |
| Dialysis | 268 (2.75) | 32 (0.92) | 99 (4.76) | 137 (3.26) | <0.001 |
| Vasopressor | 387 (3.97) | 122 (3.51) | 85 (4.08) | 180 (4.28) | 0.214 |
| Antibiotics | 727 (7.45) | 184 (5.29) | 167 (8.02) | 376 (8.95) | <0.001 |
| Outcomes |  |  |  |  |  |
| Hospital death | 1038 (10.64) | 405 (11.65) | 212 (10.19) | 421 (10.02) | 0.052 |
| ICU death | 605 (6.20) | 225 (6.47) | 133 (6.39) | 247 (5.88) | 0.514 |
| Hospital information | |  |  |  |  |
| Bed capacity |  |  |  |  | <0.001 |
| >= 500 | 4844 (49.64) | 2931 (84.35) | 1130 (54.30) | 783 (18.63) |  |
| <100 | 272 (2.79) | 0 (0.00) | 37 (1.78) | 235 (5.59) |  |
| 100 - 249 | 1519 (15.57) | 80 (2.30) | 144 (6.92) | 1295 (30.82) |  |
| 250 - 499 | 2195 (22.49) | 173 (4.98) | 544 (26.14) | 1478 (35.17) |  |
| unknown | 928 (9.51) | 291 (8.37) | 226 (10.86) | 411 (9.78) |  |
| Teaching status | 3462 (35.48) | 2337 (67.25) | 591 (28.40) | 534 (12.71) | <0.001 |

Data are presented as number (percentage) or median [interquartile range].

APACHE IV, Acute Physiology and Chronic Health Evaluation IV; CKD, Chronic kidney disease; COPD, Chronic Obstructive Pulmonary Disease.

**Supplementary Table 3. Demographic, clinical, and admitted hospital information characteristics of patients admitted with ACS.**

|  | Overall | Good triage | Bad triage | General ICU | p |
| --- | --- | --- | --- | --- | --- |
| n | 8343 | 4289 | 620 | 3434 |  |
| Age | 65.00 [55.00, 74.00] | 64.00 [55.00, 73.00] | 66.50 [56.00, 76.00] | 66.00 [56.00, 75.00] | <0.001 |
| Gender, Male | 5505 (65.98) | 2855 (66.57) | 405 (65.32) | 2245 (65.38) | 0.513 |
| APACHE IV | 39.00 [30.00, 51.00] | 39.00 [30.00, 50.00] | 38.00 [29.00, 52.25] | 40.00 [30.00, 51.00] | 0.295 |
| Comorbidities |  |  |  |  |  |
| Hypertension | 1321 (15.83) | 723 (16.86) | 62 (10.00) | 536 (15.61) | <0.001 |
| Diabetes | 934 (11.20) | 470 (10.96) | 42 (6.77) | 422 (12.29) | <0.001 |
| CKD | 580 (6.95) | 289 (6.74) | 27 (4.35) | 264 (7.69) | 0.008 |
| COPD | 296 (3.55) | 139 (3.24) | 20 (3.23) | 137 (3.99) | 0.19 |
| Heart failure | 515 (6.17) | 235 (5.48) | 37 (5.97) | 243 (7.08) | 0.015 |
| Cancer | 76 (0.91) | 36 (0.84) | 5 (0.81) | 35 (1.02) | 0.682 |
| ICU intervention |  |  |  |  |  |
| Ventilation | 833 (9.98) | 453 (10.56) | 61 (9.84) | 319 (9.29) | 0.178 |
| Dialysis | 144 (1.73) | 79 (1.84) | 19 (3.06) | 46 (1.34) | 0.007 |
| Vasopressor | 441 (5.29) | 278 (6.48) | 26 (4.19) | 137 (3.99) | <0.001 |
| Antibiotics | 398 (4.77) | 173 (4.03) | 35 (5.65) | 190 (5.53) | 0.005 |
| Outcomes |  |  |  |  |  |
| Hospital death | 328 (3.93) | 164 (3.82) | 34 (5.48) | 130 (3.79) | 0.118 |
| ICU death | 209 (2.51) | 105 (2.45) | 21 (3.39) | 83 (2.42) | 0.343 |
| Hospital information |  |  |  |  |  |
| Bed capacity |  |  |  |  | <0.001 |
| >= 500 | 3179 (38.10) | 2648 (61.74) | 233 (37.58) | 298 (8.68) |  |
| <100 | 280 (3.36) | 12 (0.28) | 42 (6.77) | 226 (6.58) |  |
| 100 - 249 | 1799 (21.56) | 501 (11.68) | 91 (14.68) | 1207 (35.15) |  |
| 250 - 499 | 2130 (25.53) | 715 (16.67) | 182 (29.35) | 1233 (35.91) |  |
| unknown | 955 (11.45) | 413 (9.63) | 72 (11.61) | 470 (13.69) |  |
| Teaching status | 1945 (23.31) | 1582 (36.89) | 112 (18.06) | 251 (7.31) | <0.001 |

Data are presented as number (percentage) or median [interquartile range].

APACHE IV, Acute Physiology and Chronic Health Evaluation IV; CKD, Chronic kidney disease; COPD, Chronic Obstructive Pulmonary Disease.

**Supplementary Table 4. Demographic, clinical, and admitted hospital information characteristics of patients admitted with CABG.**

|  | Overall | Good triage | Bad triage | General ICU | p |
| --- | --- | --- | --- | --- | --- |
| n | 4771 | 3044 | 403 | 1324 |  |
| Age | 67.00 [60.00, 74.00] | 67.00 [60.00, 74.00] | 67.00 [59.50, 75.00] | 67.00 [60.00, 73.00] | 0.846 |
| Gender, Male | 3553 (74.47) | 2263 (74.34) | 295 (73.20) | 995 (75.15) | 0.708 |
| APACHE IV | 55.00 [44.00, 70.00] | 56.00 [45.00, 71.00] | 52.00 [41.00, 65.00] | 55.00 [45.00, 69.00] | <0.001 |
| Comorbidities |  |  |  |  |  |
| Hypertension | 801 (16.79) | 549 (18.04) | 31 (7.69) | 221 (16.69) | <0.001 |
| Diabetes | 761 (15.95) | 476 (15.64) | 56 (13.90) | 229 (17.30) | 0.194 |
| CKD | 400 (8.38) | 269 (8.84) | 11 (2.73) | 120 (9.06) | <0.001 |
| COPD | 163 (3.42) | 98 (3.22) | 7 (1.74) | 58 (4.38) | 0.023 |
| Heart failure | 163 (3.42) | 113 (3.71) | 15 (3.72) | 35 (2.64) | 0.19 |
| Cancer | 23 (0.48) | 16 (0.53) | 2 (0.50) | 5 (0.38) | 0.809 |
| ICU intervention |  |  |  |  |  |
| Ventilation | 3868 (81.07) | 2384 (78.32) | 356 (88.34) | 1128 (85.20) | <0.001 |
| Dialysis | 106 (2.22) | 66 (2.17) | 9 (2.23) | 31 (2.34) | 0.938 |
| Vasopressor | 1605 (33.64) | 1102 (36.20) | 155 (38.46) | 348 (26.28) | <0.001 |
| Antibiotics | 1218 (25.53) | 771 (25.33) | 105 (26.05) | 342 (25.83) | 0.911 |
| Outcomes |  |  |  |  |  |
| Hospital death | 84 (1.76) | 55 (1.81) | 2 (0.50) | 27 (2.04) | 0.113 |
| ICU death | 58 (1.22) | 36 (1.18) | 1 (0.25) | 21 (1.59) | 0.096 |
| Hospital information |  |  |  |  |  |
| Bed capacity |  |  |  |  | <0.001 |
| >= 500 | 2354 (49.34) | 1896 (62.29) | 323 (80.15) | 135 (10.20) |  |
| <250 | 669 (14.02) | 322 (10.58) | 6 (1.49) | 341 (25.76) |  |
| 250 - 499 | 1227 (25.72) | 507 (16.66) | 74 (18.36) | 646 (48.79) |  |
| unknown | 521 (10.92) | 319 (10.48) | 0 (0.00) | 202 (15.26) |  |
| Teaching status | 1502 (31.48) | 1402 (46.06) | 0 (0.00) | 100 (7.55) | <0.001 |

Data are presented as number (percentage) or median [interquartile range].

APACHE IV, Acute Physiology and Chronic Health Evaluation IV; CKD, Chronic kidney disease; COPD, Chronic Obstructive Pulmonary Disease.

**Supplementary Table 5. Demographic, clinical, and admitted hospital information characteristics of patients admitted with PNA/Other respiratory diseases.**

|  | Overall | Good triage | Bad triage | General ICU | p |
| --- | --- | --- | --- | --- | --- |
| n | 12547 | 1892 | 2749 | 7906 |  |
| Age | 66.00 [55.00, 77.00] | 65.00 [54.00, 76.00] | 66.00 [55.00, 77.00] | 67.00 [55.00, 78.00] | 0.019 |
| Gender, Male | 6452 (51.42) | 931 (49.21) | 1472 (53.55) | 4049 (51.21) | 0.012 |
| APACHE IV | 55.00 [40.00, 73.00] | 58.00 [42.75, 78.00] | 55.00 [41.00, 72.00] | 55.00 [40.00, 72.00] | <0.001 |
| Comorbidities |  |  |  |  |  |
| Hypertension | 1828 (14.57) | 211 (11.15) | 395 (14.37) | 1222 (15.46) | <0.001 |
| Diabetes | 1492 (11.89) | 219 (11.58) | 286 (10.40) | 987 (12.48) | 0.013 |
| CKD | 1188 (9.47) | 210 (11.10) | 268 (9.75) | 710 (8.98) | 0.016 |
| COPD | 2203 (17.56) | 331 (17.49) | 350 (12.73) | 1522 (19.25) | <0.001 |
| Heart failure | 1342 (10.70) | 182 (9.62) | 288 (10.48) | 872 (11.03) | 0.187 |
| Cancer | 854 (6.81) | 120 (6.34) | 163 (5.93) | 571 (7.22) | 0.047 |
| ICU intervention |  |  |  |  |  |
| Ventilation | 6519 (51.96) | 1084 (57.29) | 1402 (51.00) | 4033 (51.01) | <0.001 |
| Dialysis | 538 (4.29) | 107 (5.66) | 122 (4.44) | 309 (3.91) | 0.003 |
| Vasopressor | 1040 (8.29) | 167 (8.83) | 231 (8.40) | 642 (8.12) | 0.588 |
| Antibiotics | 5308 (42.30) | 686 (36.26) | 1086 (39.51) | 3536 (44.73) | <0.001 |
| Outcomes |  |  |  |  |  |
| Hospital death | 1682 (13.41) | 279 (14.75) | 380 (13.82) | 1023 (12.94) | 0.09 |
| ICU death | 1013 (8.07) | 160 (8.46) | 244 (8.88) | 609 (7.70) | 0.121 |
| Hospital information |  |  |  |  |  |
| Bed capacity |  |  |  |  | <0.001 |
| >= 500 | 4900 (39.05) | 1407 (74.37) | 1517 (55.18) | 1976 (24.99) |  |
| <100 | 849 (6.77) | 35 (1.85) | 64 (2.33) | 750 (9.49) |  |
| 100 - 249 | 2715 (21.64) | 117 (6.18) | 262 (9.53) | 2336 (29.55) |  |
| 250 - 499 | 2805 (22.36) | 99 (5.23) | 615 (22.37) | 2091 (26.45) |  |
| unknown | 1278 (10.19) | 234 (12.37) | 291 (10.59) | 753 (9.52) |  |
| Teaching status | 3686 (29.38) | 811 (42.86) | 1110 (40.38) | 1765 (22.32) | <0.001 |

Data are presented as number (percentage) or median [interquartile range].

APACHE IV, Acute Physiology and Chronic Health Evaluation IV; CKD, Chronic kidney disease; COPD, Chronic Obstructive Pulmonary Disease.

**Supplementary Table 6. Demographic, clinical, and admitted hospital information characteristics of patients admitted with trauma.**

|  | Overall | Good triage | Bad triage | General ICU | p |
| --- | --- | --- | --- | --- | --- |
| n | 5884 | 1093 | 1819 | 2972 |  |
| Age | 58.00 [36.00, 76.00] | 52.00 [32.00, 68.00] | 60.00 [39.00, 78.00] | 59.00 [37.00, 77.00] | <0.001 |
| Gender, Male | 3746 (63.66) | 760 (69.53) | 1145 (62.95) | 1841 (61.94) | <0.001 |
| APACHE IV | 45.00 [31.00, 61.00] | 46.00 [31.00, 64.00] | 43.00 [31.00, 59.00] | 45.00 [32.00, 62.00] | 0.002 |
| Comorbidities |  |  |  |  |  |
| Hypertension | 478 (8.12) | 91 (8.33) | 136 (7.48) | 251 (8.45) | 0.474 |
| Diabetes | 238 (4.04) | 37 (3.39) | 59 (3.24) | 142 (4.78) | 0.015 |
| CKD | 198 (3.37) | 32 (2.93) | 51 (2.80) | 115 (3.87) | 0.094 |
| COPD | 120 (2.04) | 24 (2.20) | 27 (1.48) | 69 (2.32) | 0.127 |
| Heart failure | 71 (1.21) | 7 (0.64) | 26 (1.43) | 38 (1.28) | 0.148 |
| Cancer | 45 (0.76) | 12 (1.10) | 14 (0.77) | 19 (0.64) | 0.33 |
| ICU intervention |  |  |  |  |  |
| Ventilation | 1876 (31.88) | 478 (43.73) | 503 (27.65) | 895 (30.11) | <0.001 |
| Dialysis | 63 (1.07) | 12 (1.10) | 21 (1.15) | 30 (1.01) | 0.89 |
| Vasopressor | 409 (6.95) | 99 (9.06) | 109 (5.99) | 201 (6.76) | 0.006 |
| Antibiotics | 741 (12.59) | 125 (11.44) | 174 (9.57) | 442 (14.87) | <0.001 |
| Outcomes |  |  |  |  |  |
| Hospital death | 431 (7.32) | 88 (8.05) | 134 (7.37) | 209 (7.03) | 0.541 |
| ICU death | 292 (4.96) | 65 (5.95) | 83 (4.56) | 144 (4.85) | 0.229 |
| Hospital information | |  |  |  |  |
| Bed capacity |  |  |  |  | <0.001 |
| >= 500 | 2970 (50.48) | 987 (90.30) | 1195 (65.70) | 788 (26.51) |  |
| <100 | 155 (2.63) | 59 (5.40) | 13 (0.71) | 83 (2.79) |  |
| 100 - 249 | 1006 (17.10) | 25 (2.29) | 266 (14.62) | 715 (24.06) |  |
| 250 - 499 | 1410 (23.96) | 16 (1.46) | 286 (15.72) | 1108 (37.28) |  |
| unknown | 343 (5.83) | 6 (0.55) | 59 (3.24) | 278 (9.35) |  |
| Teaching status | 1971 (33.50) | 629 (57.55) | 688 (37.82) | 654 (22.01) | <0.001 |

Data are presented as number (percentage) or median [interquartile range].

APACHE IV, Acute Physiology and Chronic Health Evaluation IV; CKD, Chronic kidney disease; COPD, Chronic Obstructive Pulmonary Disease.

**Supplementary Table 7. regression coefficients from parallel multiple mediation analysis in CVA subgroup.**

| Variable | Hospital Mortality (Unadjusted) | Mechanical Ventilation | Dialysis | Vasopressor Use | Antibiotics Use | Hospital Mortality (Adjusted) |
| --- | --- | --- | --- | --- | --- | --- |
| Intercept | -2.654*** (0.610) | -2.718*** (0.731) | -18.780 (590.196) | -16.621 (392.444) | -3.098*** (0.738) | -2.650*** (0.641) |
| Age | 0.021*** (0.003) | -0.004 (0.002) | -0.028*** (0.006) | -0.012** (0.004) | -0.009** (0.003) | 0.031*** (0.003) |
| Gender (Male) | 0.202* (0.087) | 0.023 (0.070) | -0.152 (0.204) | 0.036 (0.142) | -0.083 (0.112) | 0.241* (0.097) |
| Hypertension | -0.350** (0.107) | -0.262** (0.085) | 0.530* (0.244) | -0.321 (0.174) | 0.358** (0.127) | -0.248* (0.118) |
| Diabetes | 0.217 (0.161) | 0.094 (0.137) | -0.347 (0.273) | 0.018 (0.290) | 0.778*** (0.162) | 0.246 (0.180) |
| CKD | 0.157 (0.183) | -0.062 (0.159) | 3.529*** (0.235) | -0.357 (0.347) | 0.278 (0.191) | 0.309 (0.214) |
| COPD | 0.119 (0.319) | -0.162 (0.296) | -0.619 (0.667) | -0.091 (0.599) | 0.283 (0.349) | 0.332 (0.340) |
| Heart Failure | 0.266 (0.267) | 0.590** (0.208) | -0.211 (0.441) | 0.661 (0.384) | 0.646* (0.258) | 0.053 (0.291) |
| Cancer | -0.496 (0.397) | -0.646 (0.337) | -0.490 (0.772) | -0.161 (0.593) | 0.632 (0.332) | -0.244 (0.429) |
| Teaching status | -0.033 (0.104) | -0.281*** (0.084) | 0.177 (0.272) | 0.089 (0.174) | -0.200 (0.138) | 0.128 (0.114) |
| Bed capacity |  |  |  |  |  |  |
| ≥500 beds | 0.626 (0.613) | 1.544* (0.732) | 13.298 (590.196) | 13.477 (392.444) | 0.157 (0.743) | -0.051 (0.645) |
| 100-249 beds | -0.330 (0.682) | 0.826 (0.758) | 14.037 (590.196) | 12.317 (392.444) | 0.277 (0.771) | -0.773 (0.721) |
| 250-499 beds | 0.146 (0.622) | 1.079 (0.736) | 14.208 (590.196) | 13.386 (392.444) | 0.361 (0.745) | -0.288 (0.654) |
| Unknown | 0.399 (0.625) | 1.195 (0.739) | 13.785 (590.196) | 12.859 (392.444) | 0.381 (0.755) | -0.068 (0.658) |
| ICU Triage (concordant vs discordant) | -0.014 (0.051) | 0.037 (0.042) | -0.376** (0.120) | -0.182* (0.084) | -0.071 (0.064) | -0.010 (0.056) |
| Mediators |  |  |  |  |  |  |
| Mechanical Ventilation | - | - | - | - | - | 2.523*** (0.102) |
| Dialysis | - | - | - | - | - | -0.284 (0.336) |
| Vasopressor Use | - | - | - | - | - | 0.628*** (0.176) |
| Antibiotics Use | - | - | - | - | - | -0.280 (0.176) |
| Model Fit Indices |  |  |  |  |  |  |
| McFadden's R² | 0.026 | 0.012 | 0.354 | 0.017 | 0.036 | 0.218 |
| Nagelkerke's R² | 0.036 | 0.018 | 0.380 | 0.020 | 0.045 | 0.281 |
| AIC | 3802.371 | 5249.282 | 831.468 | 1768.318 | 2553.997 | 3066.331 |
| BIC | 3901.711 | 5348.622 | 930.807 | 1867.657 | 2653.336 | 3192.161 |

Values represent regression coefficients with standard errors in parentheses. *p < .05, **p < .01, ***p < .001. Reference categories: Female for gender, No for comorbidities, Non-teaching hospital, <100 beds for hospital size, discordant ICU triage. All continuous variables are grand-mean centered.*

**Supplementary Table 8. regression coefficients from parallel multiple mediation analysis in trauma subgroup.**

| Variable | Hospital Mortality (Unadjusted) | Mechanical Ventilation | Dialysis | Vasopressor Use | Antibiotics Use | Hospital Mortality (Adjusted) |
| --- | --- | --- | --- | --- | --- | --- |
| Intercept | -4.489*** (1.017) | -4.932*** (1.011) | -19.759 (1204.385) | -17.999 (463.470) | -4.612*** (1.016) | -3.698*** (1.034) |
| Age | 0.015*** (0.003) | -0.019*** (0.002) | -0.020 (0.010) | -0.005 (0.004) | -0.017*** (0.003) | 0.027*** (0.004) |
| Gender (Male) | 0.213 (0.155) | 0.391*** (0.092) | -0.351 (0.394) | 0.334* (0.168) | -0.050 (0.139) | -0.073 (0.171) |
| Hypertension | -0.237 (0.272) | -0.020 (0.168) | -0.392 (0.596) | -0.032 (0.274) | 0.842*** (0.199) | -0.121 (0.296) |
| Diabetes | 0.043 (0.377) | -0.448 (0.268) | 0.029 (0.711) | 0.362 (0.363) | 0.941*** (0.272) | 0.303 (0.424) |
| CKD | 0.694* (0.334) | 0.284 (0.260) | 3.953*** (0.494) | 0.220 (0.404) | -0.029 (0.375) | 0.715 (0.393) |
| COPD | 0.958* (0.387) | 0.334 (0.315) | 0.979 (0.811) | 1.101** (0.388) | 1.334*** (0.333) | 0.549 (0.472) |
| Heart Failure | -0.511 (0.666) | 1.205** (0.385) | -0.080 (0.939) | 1.290** (0.476) | 0.596 (0.471) | -1.710* (0.771) |
| Cancer | -14.248 (462.862) | 0.344 (0.425) | 1.551 (1.131) | 0.010 (0.747) | 0.963* (0.478) | -14.400 (419.251) |
| Teaching status | 0.277 (0.154) | 0.009 (0.091) | -0.555 (0.438) | 0.358* (0.162) | -0.417** (0.141) | 0.298 (0.170) |
| Bed capacity |  |  |  |  |  |  |
| ≥500 beds | 1.777 (1.018) | 4.011*** (1.011) | 15.061 (1204.385) | 14.976 (463.470) | 2.353* (1.016) | 0.586 (1.041) |
| 100-249 beds | 0.653  (1.076) | 3.583***  (1.021) | 14.49 (1204.385) | 14.641 (463.471) | 1.066 (1.073) | -0.615 (1.105) |
| 250-499 beds | 1.454 (1.045) | 3.986*** (1.020) | 15.548 (1204.385) | 14.886 (463.470) | 3.344** (1.026) | 0.235 (1.073) |
| Unknown | 1.728 (1.117) | 3.610*** (1.057) | 0.525 (1782.321) | 15.206 (463.471) | 2.434* (1.108) | 0.545 (1.162) |
| ICU Triage (concordant vs discordant) | 0.012 (0.077) | 0.318*** (0.045) | 0.087 (0.215) | 0.172* (0.079) | 0.195** (0.072) | -0.202* (0.086) |
| Mediators |  |  |  |  |  |  |
| Mechanical Ventilation | - | - | - | - | - | 2.027*** (0.184) |
| Dialysis | - | - | - | - | - | -0.150 (0.514) |
| Vasopressor Use | - | - | - | - | - | 1.891*** (0.199) |
| Antibiotics Use | - | - | - | - | - | -0.562* (0.258) |
| Model Fit Indices |  |  |  |  |  |  |
| McFadden's R² | 0.041 | 0.083 | 0.217 | 0.038 | 0.088 | 0.225 |
| Nagelkerke's R² | 0.053 | 0.139 | 0.228 | 0.048 | 0.117 | 0.274 |
| AIC | 1534.573 | 3443.492 | 312.831 | 1471.303 | 1786.999 | 1254.414 |
| BIC | 1624.222 | 3533.141 | 402.480 | 1560.952 | 1876.648 | 1367.969 |

Values represent regression coefficients with standard errors in parentheses. *p < .05, **p < .01, ***p < .001. Reference categories: Female for gender, No for comorbidities, Non-teaching hospital, <100 beds for hospital size, discordant ICU triage. All continuous variables are grand-mean centered.*
